# Job Analysis and Curriculum Design of South Korean Animal-Assisted Therapy Specialists Using DACUM

**DOI:** 10.3390/ani14131943

**Published:** 2024-06-30

**Authors:** Soo Jeong Choi, Jin Soo Han

**Affiliations:** 1 Department of Bio and Healing Convergence, Graduate School, Konkuk University, Seoul 05029, Republic of Korea; somdodam@konkuk.ac.kr; 2 Department of Laboratory Animal Medicine, Institute for the 3Rs & Animal Welfare, College of Veterinary Medicine, Konkuk University, Seoul 05029, Republic of Korea

**Keywords:** animal-assisted intervention, animal-assisted therapy, animal-assisted therapist, job analysis, DACUM method

## Abstract

**Simple Summary:**

The animal-assisted therapy (AAT) is a goal-oriented, planned, and structured treatment intervention directed and delivered by professionals. AAT is in the spotlight as a beneficial therapy for humans. However, there is a lack of research on this type of job or the education that these professionals should receive. This study aimed to analyze the job of the person executing AAT, design the curriculum, and provide discussions and suggestions for future research directions.

**Abstract:**

This study analyzed the jobs of animal-assisted therapy specialists using the Development of a Curriculum (DACUM) technique, a job analysis method for the duties and tasks performed in a specific job. It derived nine duties and 54 tasks through a verification process. In addition, by analyzing the knowledge, skills, and attitudes according to the task, the duties of animal-assisted therapy specialists were derived with 37 knowledge points (K), 32 skills (S), and 46 attitudes (A). The curriculum was designed based on the results derived from the job analysis. The final derived subjects were “understanding the counselee”, “clinical practice”, “therapy-assisted animal management”, “case conceptualization”, “psychological test and evaluation”, “program development”, “understanding and practice of counseling psychology”, “animal-assisted intervention introduction”, “evaluation analysis and report”, “case study and practice”, “case guidance and management”, “training and behavior”, and “animal welfare”. These results can improve the professionalism of animal-assisted therapy specialists and the overall quality of the therapy site.

## 1. Introduction

Animal-assisted interventions (AAIs) have developed significantly over the past half century [1]. In 1950, child psychologist Boris Levinson discovered that using dogs to facilitate communication between therapists and patients could be beneficial for the treatment, which led Levinson to coin the term Pet Therapy in 1964 [2] and develop the Pet-Facilitated Psychotherapy and Pet-Oriented Child Psychotherapy theories [3]. Scientific and systematic animal-assisted therapy was initiated by the National Institutes of Health (NIH) in 1988. According to a white paper by IAHAIO, the International Association of Human–Animal Interaction Organizations, AAIs are a goal-oriented and structured intervention that intentionally include or integrate animals into health, education, and services aimed at the therapeutic benefit of humans. Animal-assisted therapy (AAT), one of the subcategories of AAIs, is a goal-oriented, planned, and structured treatment intervention directed and delivered by a professional. Professionals performing AAT should have knowledge of animal behavior, needs, health indicators, and stress control, focusing on improving a particular subject’s physical, cognitive, behavioral, and socio-emotional functions [4]. AAT is being implemented in response to various symptoms and for different conditions, including autism [5,6,7], cognitive impairment and mobility [8], depression, anxiety [9], and dementia [10,11], and is being carried out in various environments, including mental health facilities [12,13], nursing homes [14,15], and hospitals [16]. Therefore, animal-assisted therapy specialists who perform AAT should be professionals with specialized knowledge in human physical, cognitive, behavioral, and social-emotional aspects and animal behavior, needs, health, and stress [4]. Despite the high level of expertise required for this job beyond simple interactions with animals, research on the duties performed by animal-assisted therapy specialists is still lacking.

The Developing a Curriculum (DACUM) technique is a job analysis method used to analyze the duties and tasks performed by a particular profession to develop a curriculum. The DACUM technique uses group interaction, synergy, and brainstorming. Compared to other techniques, it can be performed economically in a relatively short amount of time. This technique more systematically derives the characteristics of the job, knowledge, skills, and attitudes required through workshops with experienced experts [17,18,19]. DACUM was used to analyze jobs in various occupations, such as horticultural therapists (currently, welfare therapists) [20], occupational therapists [21], physical therapists [22], and forest prenatal care instructors [23] to increase job efficiency and professionalism by specifying and systematizing job contents. In addition, various occupations, such as nurses [24], professional counselors [25], and plant protection technicians [26], were analyzed through job analysis using DACUM to lay the foundation for these jobs and systematize them. All the jobs that were analyzed were newly created according to the trends of the times; thus, they tried to systematically operate them based on an understanding of and the evidence from actual work in the field through a job analysis. Currently, there are various definitions of animal-assisted interventions (AAIs), AAT, and animal-assisted education (AAE) in South Korea and abroad, and the problem is that they are inconsistent. In other areas, such as occupational therapy and physical therapy, all forms of intervention, including those that involve animals, are classified as animal-assisted therapy. This broad classification leads to potential problems because the corresponding programs can proceed with a lack of understanding of human–animal interactions and without evaluating animal behavior or temperament.

Currently, in South Korea, a degree or license is required to work as an animal-assisted therapy specialist, and individuals must undergo sufficient education. However, each institution implements programs with different names and contents, which are neither internationalized nor standardized. Consequently, a curriculum for actual activities in the field has not been developed. Moreover, there is a lack of research or data analysis on the duties of animal-assisted therapy specialists. This study aimed to analyze the duties of animal-assisted therapy specialists and design a curriculum using the DACUM technique. By enhancing the understanding of the role of animal-assisted therapy specialists through a job analysis, we anticipate improvements in the efficiency and quality of treatment in the clinical field, as well as in the professionalism of practitioners. Additionally, we expect the welfare of treatment-assisting animals and clients to improve.

## 2. Materials and Methods

### 2.1. DACUM Commission

To form the DACUM committee, among the animal-assisted therapy specialists operating across the country, those with excellent technical work ability, job representation, communication ability, full-time work, and unbiased tendencies were selected as the panel according to the panel selection criteria [19]. Eight people were selected for the DACUM panel among ten candidates who agreed to participate. The workshop consisted of one professional analyst, one recorder, and one observer to help facilitate and analyze the workshop.

### 2.2. Process of DACUM Analysis

The schedule, location, time, and principles with which the panel had to comply were sent to the panelists by e-mail to familiarize them with DACUM. The workshop lasted for 10 h. In the first hour, an orientation was conducted through an analyst to enhance the panel’s understanding of the DACUM workshop. Seats were placed around the analyst such that everyone could see and talk to each other, and the recorder was placed in the seat closest to the analyst. Sticky walls were attached to the wall, and duties, tasks, knowledge, skills, and attitudes were separated by color and attached to the wall. The panel freely discussed their opinions and changed the position of the papers whenever their opinions were revised. The recorder recorded all the meeting contents in real time using a laptop, which were recorded for future review.

### 2.3. DACUM Analysis Verification Process

#### 2.3.1. First Verification

The results derived from the DACUM workshop were sent to the panelists via e-mail and they were asked to review and provide suggestions for modifications about the duties, tasks, knowledge, skills, and attitudes. One week later, the revised DACUM chart was sent by e-mail for a second time, and the revised contents were returned two days later. Therefore, the first verification step was performed twice.

#### 2.3.2. Second Verification

The animal-assisted therapy specialist DACUM chart, developed after two first-line validations, was sent to a three-member secondary validation committee, and a reply was received four days later. The second verification consisted mainly of working-level staff in the clinical field. This was performed to check whether there was a difference between the developed DACUM chart and the clinical field and to secure additional objective opinions.

### 2.4. Curriculum Derivation

To derive a curriculum for animal-assisted therapy specialists, three working-level staff members derived key tasks from the list of tasks and grouped the knowledge, skills, and attitudes necessary for core tasks.

## 3. Results

### 3.1. The Definition of an Animal-Assisted Therapy Specialist

The definitions of animal-assisted therapy specialists derived from the DACUM workshop are presented. Among the various definitions derived, common terms were ‘animal’, ‘person’, ‘interaction’, ‘client analysis’, ‘psychotherapy intervention’, ‘environmental composition’, ‘adaptation’, ‘recovery’, ‘improvement’, ‘rehabilitation’, and ‘development’. Two rounds of verifications were conducted, and the definition of an animal-assisted therapy specialist was finally defined as “an expert who supports the psychological and emotional recovery, social adaptation, and development of the client through psychotherapy interventions using human–animal interactions”. Animal-assisted therapy specialists (Table 1) engage in psychotherapeutic interventions by interacting with animals with various subjects in various environments. After completing the curriculum set by each institution, they acquire qualifications and work in the clinical field.

### 3.2. Duties and Tasks

Through the DACUM workshop and the two rounds of verifications, the animal-assisted therapy specialists’ duties and tasks were finally defined (Table 2). The duties were divided into nine categories: securing counselors, identifying service users, designing programs, conducting counseling, evaluating counseling, follow-up management, developing therapy specialist competency, managing therapy specialists, developing new programs, and analyzing 54 tasks.

In <A. Securing Counseling Resources>, the following steps are implemented: identify the type of service user, consult on the counseling site, identify the physical environment of the counseling site, guide institutional users, and adapt treatment-assisting animals to the counseling site. To <B. Check the Service Users>, therapists should consult with institutions and individuals and conduct reception interviews. A counseling strategy is established after conducting psychological tests and conceptualizing the case. <C. Program Design> investigates the appropriate program for the client and selects the appropriate program format. The therapist selects treatment-assisting animals, determines their duties, and establishes a physical environment that considers the clients and animals. Subsequently, a suitable program for the client is designed. In <D. Progress Consultation>, the counseling environment is checked, and the condition of the treatment-assisting animal is checked before the session. After explaining animal-assisted therapy to the service users and obtaining their consent, a relationship is formed between the client, AAT specialist, and animal and they engage in psychotherapy interventions. Different physical environments should be provided according to changes in circumstances, and measures should be taken to check stress signals from treatment-assisting animals. All session processes should be documented, and conclusions should be discussed at the end of the session. In <E. Evaluation of counseling>, the consultation results are evaluated, and a result report is prepared. In <F. Follow-Up Management>, the client’s current status is identified, and additional counseling is decided. In addition, the results of follow-up management are documented. In <G. Development of the AAT Specialist Competency>, therapists learn the latest information in related fields, receive supervision, and participate in case-study meetings. They attend academic meetings and publish case results in academic journals. AAT specialists should have self-awareness and self-care skills; also, they must engage in self-promotion and marketing. In <H. Therapy-Assisting Animal Care>, the characteristics of treatment-assisting animals must be identified, and treatment-assisting animals must be educated and certified. Additionally, veterinary management and animal welfare must be practiced throughout all processes. Therefore, management plans for retirement of treatment-assisting animals should be established . In <I. Development of New Programs>, first, service users are analyzed, and then the purpose and goal of the program are set. A program suitable for service users is also developed by collecting and analyzing prior programs. The program’s validity is checked, implemented, and applied. The results are analyzed and their significance is checked. The program is revised and reviewed, and the development plan is documented. Animal-assisted therapy specialists who know their job will provide high-quality treatment services to clients and have a systematic structure for carrying out the work. In addition, the welfare and well-being of treatment-assisting animals, which are partners who work together with the therapist, can be improved through <H. Therapy-Assisting Animal Care>, which are areas that can be missed due to a focus on clients.

### 3.3. Knowledge, Skills, and Attitudes

Knowledge (K), skills (S), and attitudes (A) define the knowledge, skills, and attitudes necessary to perform each task. Knowledge is defined as the principles one needs to know to perform the job, such as understanding the basics of animal behavior, training, basic veterinary medicine, companion animal science, basic hygiene, AAT animal welfare, animal ethics and bioethics, animal protection laws, education systems and policies, welfare policies, general knowledge of animal-assisted interventions, knowledge of the effectiveness of AAIs, knowledge of AAI programs, knowledge of AAI objectives, budget planning, documentation, human zoology, recent trends, complementary medicine, psychological theory, psychological reality, counseling ethics, case conceptualization, guardian counseling, abnormal psychological psychology, developmental psychology, clients’ understanding, statistics, evaluation methods, thesis writing, case study methods, psychological tests, evaluation tools, and emergencies.

Skills refer to personality traits, psychological skills, and basic postures that must be used to perform a task. They are derived from the reception interview ability, client analysis ability, client information collection ability, treatment program development ability, therapeutic environment construction ability, counseling technique ability, animal dynamic utilization ability, administrative procedure ability, consultation ability, service regulation identification ability, physical environment standard identification ability, logical thinking ability, observation ability, work documentation ability, ability to use evaluation tools, literature data collection and analysis ability, ability to forms insight, handling ability, treatment-assisting animal health and hygiene management ability, self-reflection and self-analysis ability, AAT specialist stress and emotion management ability, crisis management ability, medical aid ability, and auxiliary device operation ability.

Attitudes are states of preparation for a response to an object based on individual perceptions and emotions. Attitudes refer to internal tendencies to react, even if they do not result in actual actions. The attitudes were defined as determined, cooperative, neutral, dispassionate, objective, reliable, thoughtful, broad-minded, gives unconditional regard, honest, provides careful concern, kind, patient, humorous, gentle, careful, sensitive, detailed, has a positive attitude, has affinity, receptive, friendly, humanity, courteous, affirmative, has transmissibility, flexible, pliable, takes the initiative and sets an example, creativity, curiosity, adventurous, progressive, has a positive mindset, self-sacrificing, leadership, trustworthy, confident, has a sense of duty, responsible, sincere, tenacious, accurate, careful, prepared, concentrated, ethical, has their own philosophy, professional, clean, has a neat appearance, manages expression, has academic fervor, and has a sense of purpose. Through the two rounds of verifications, 37 knowledge points (K), 32 skills (S), and 46 attitudes (A) were identified (Table 3).

### 3.4. Curriculum Design

**Figure 1:**
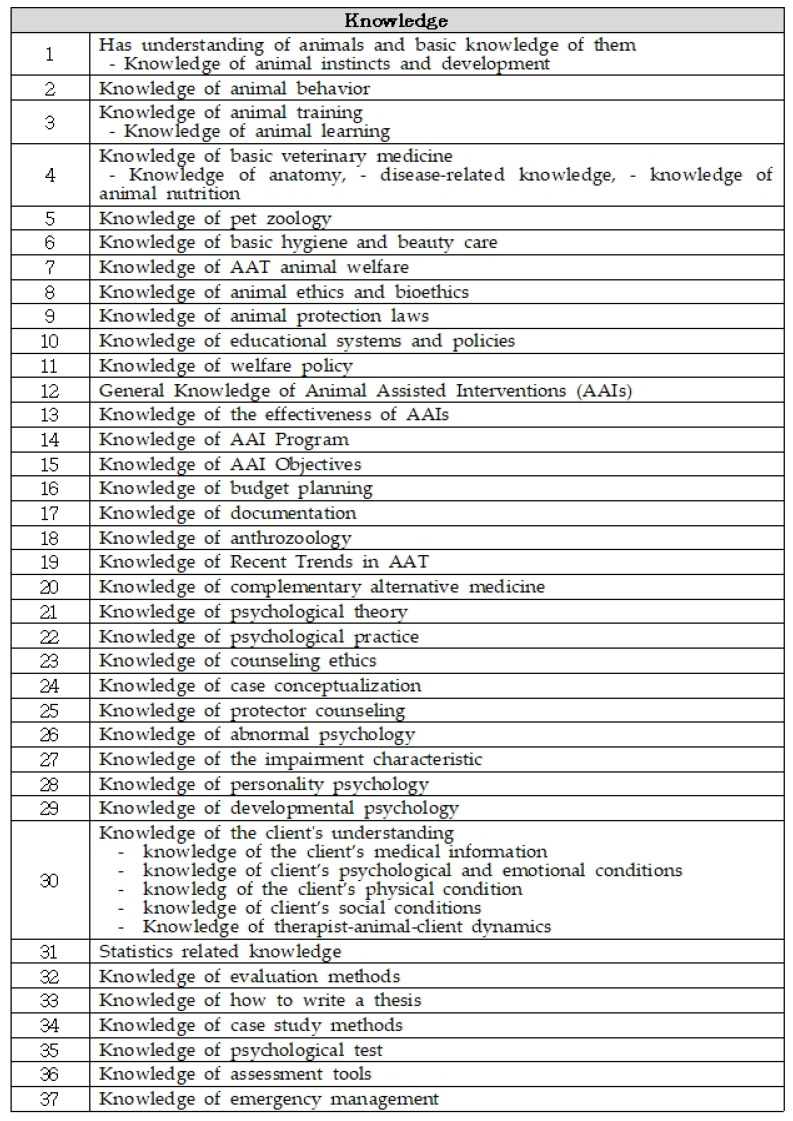

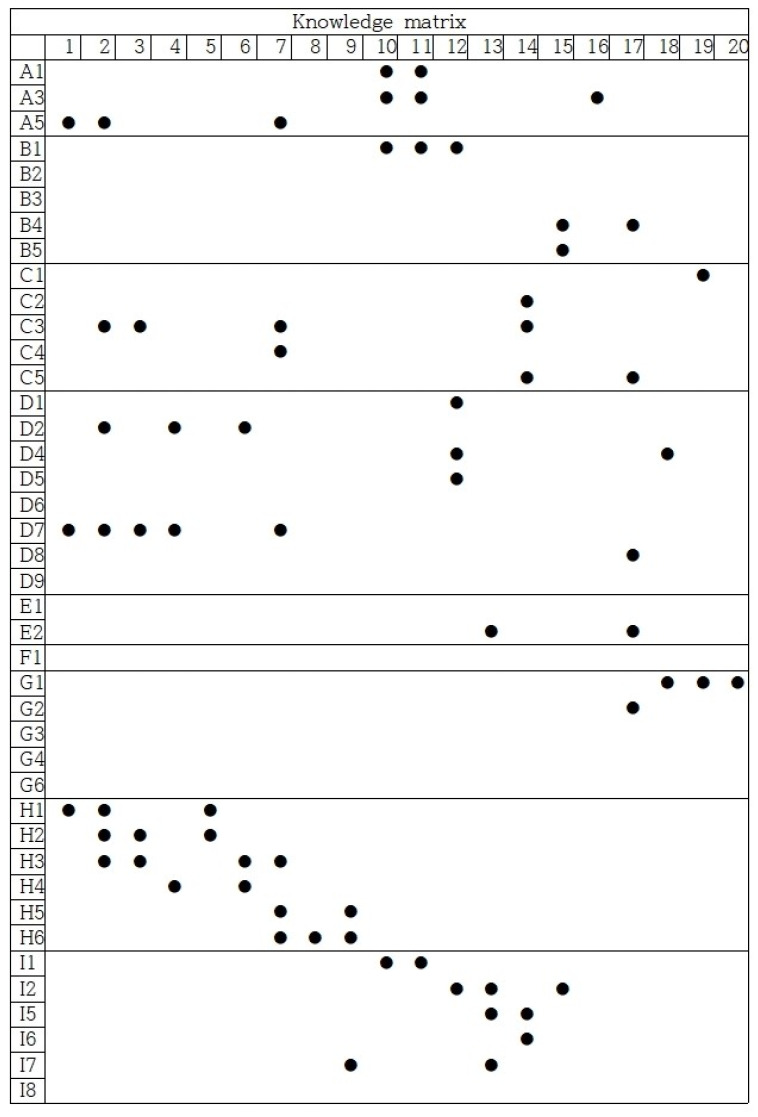

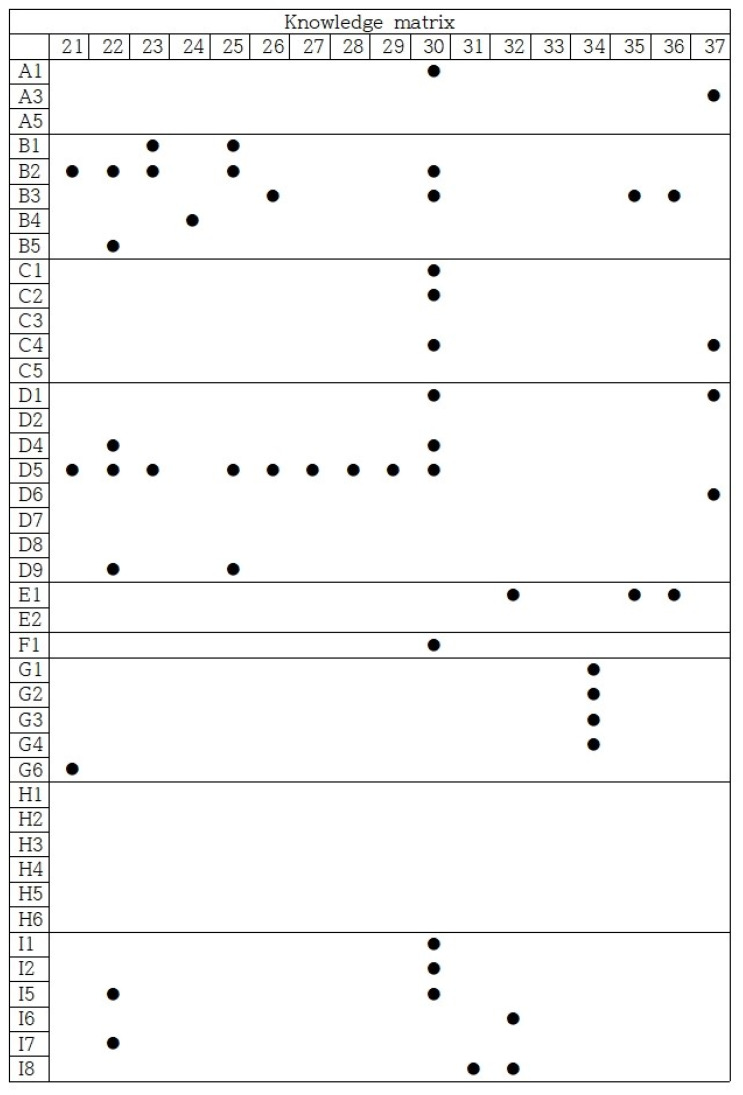

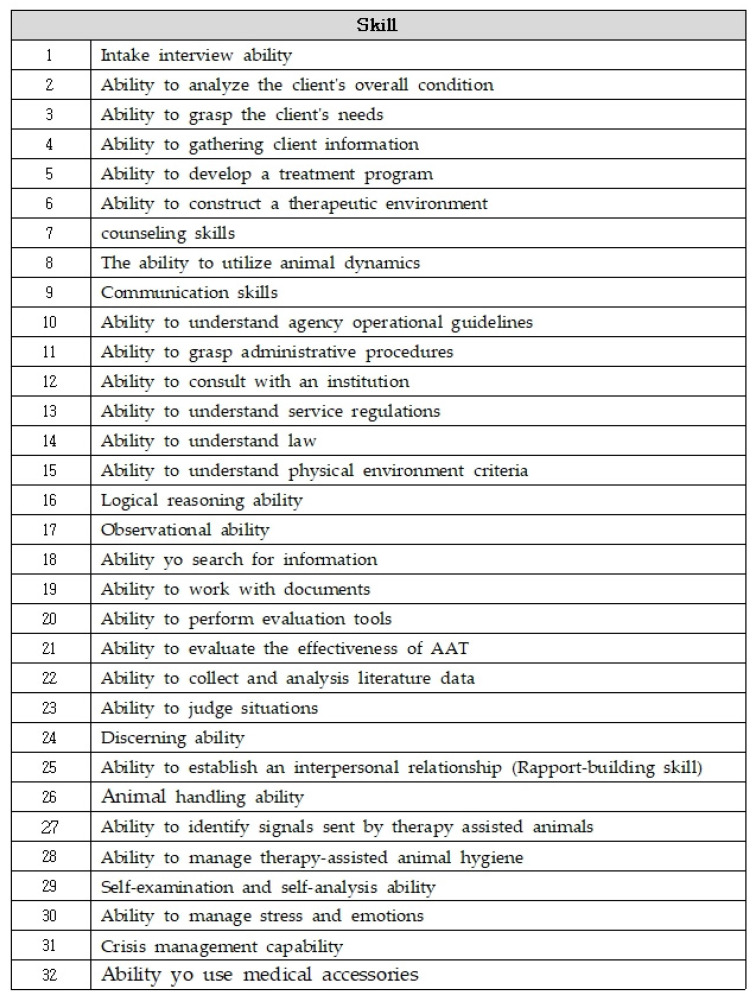

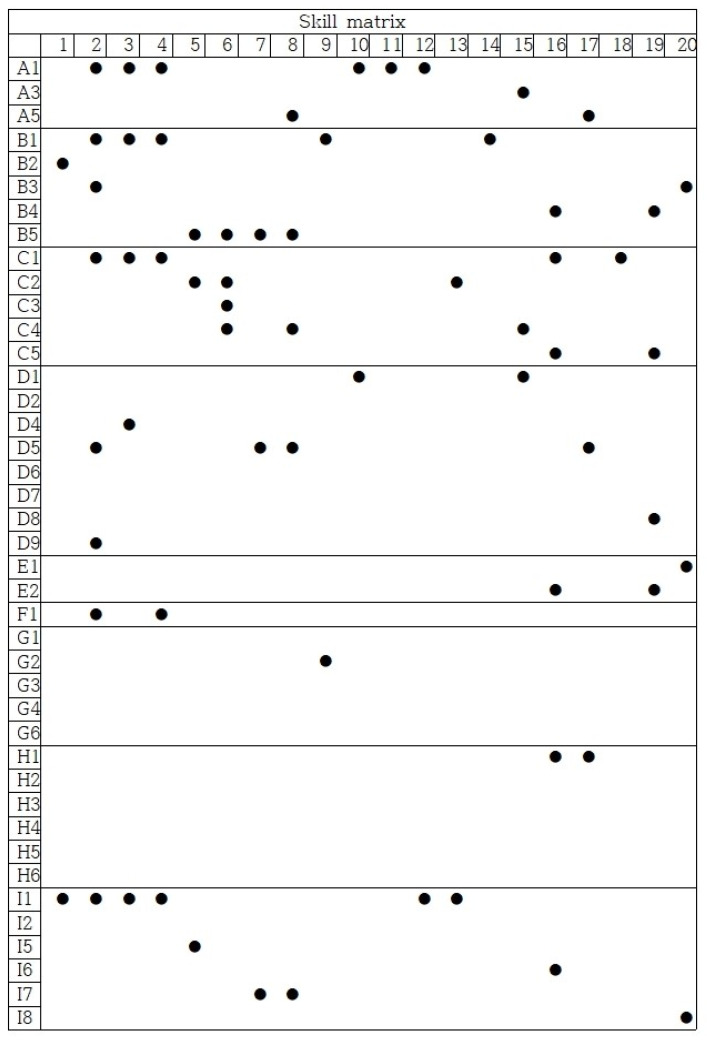

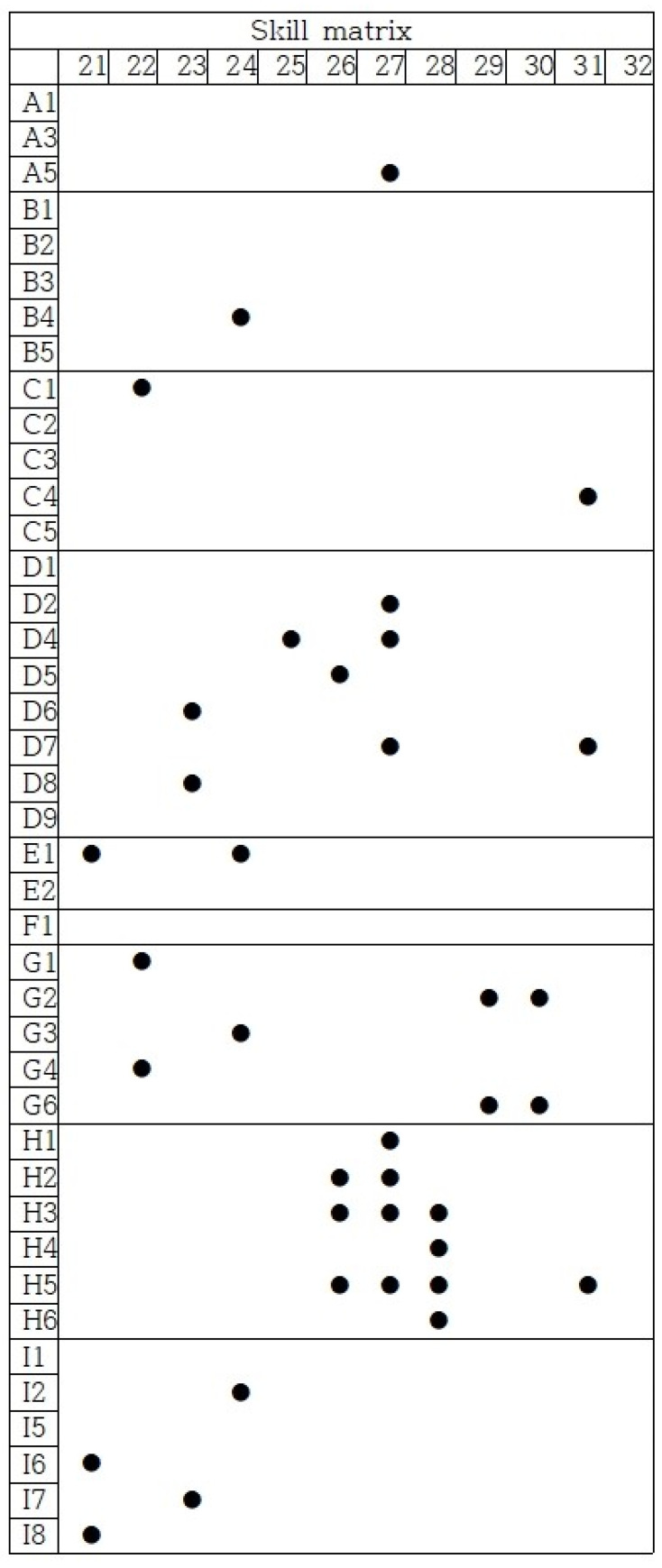

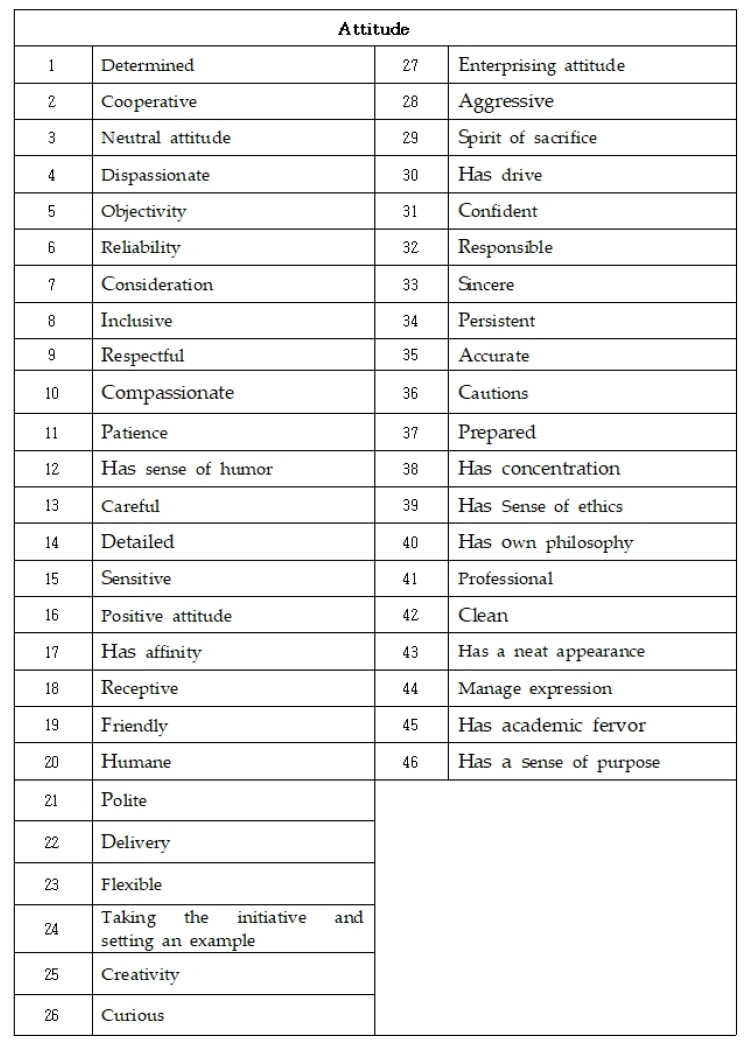

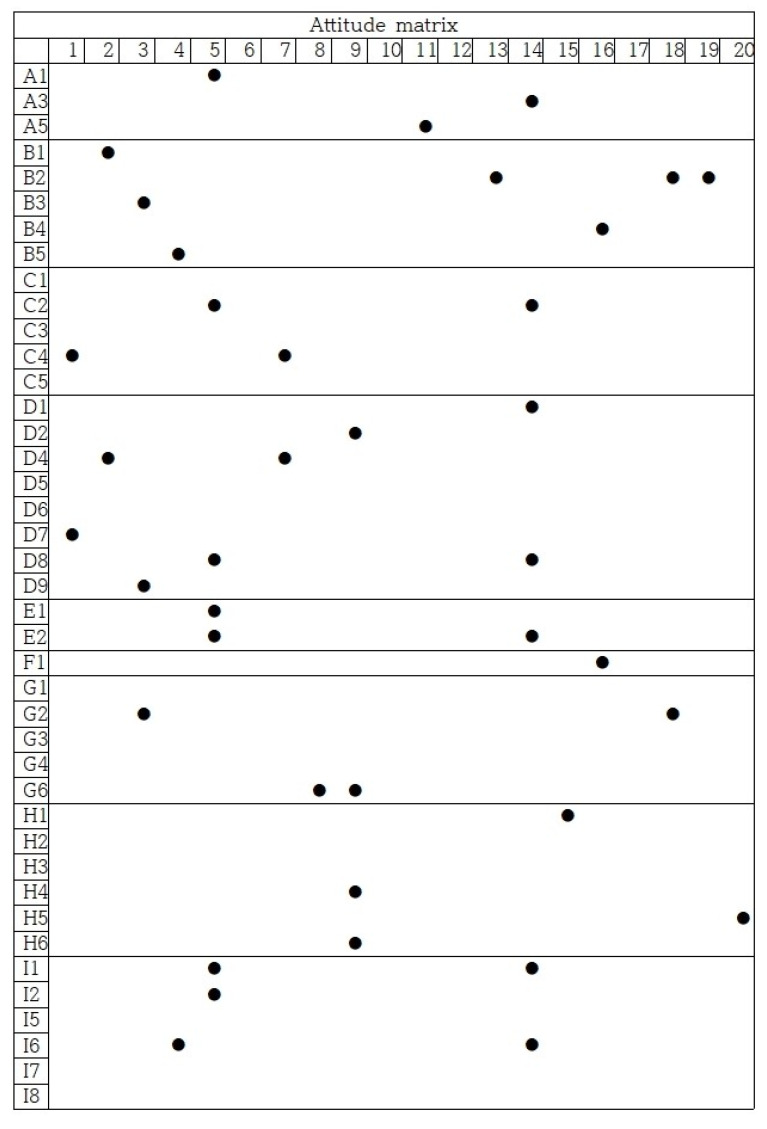

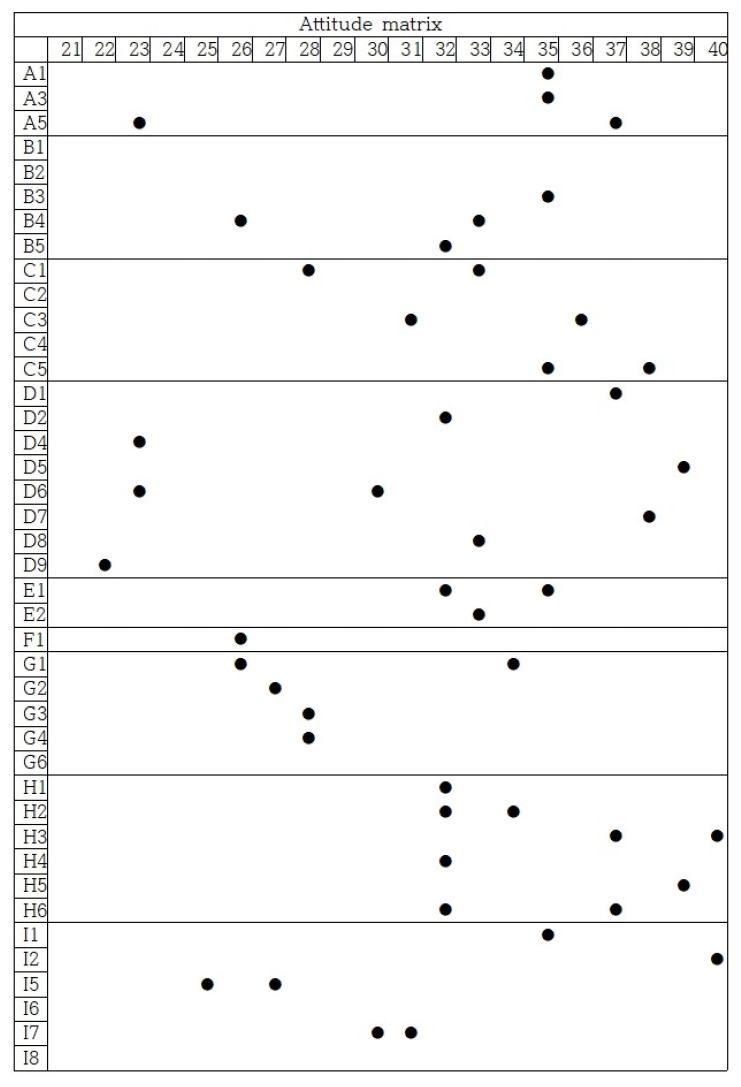

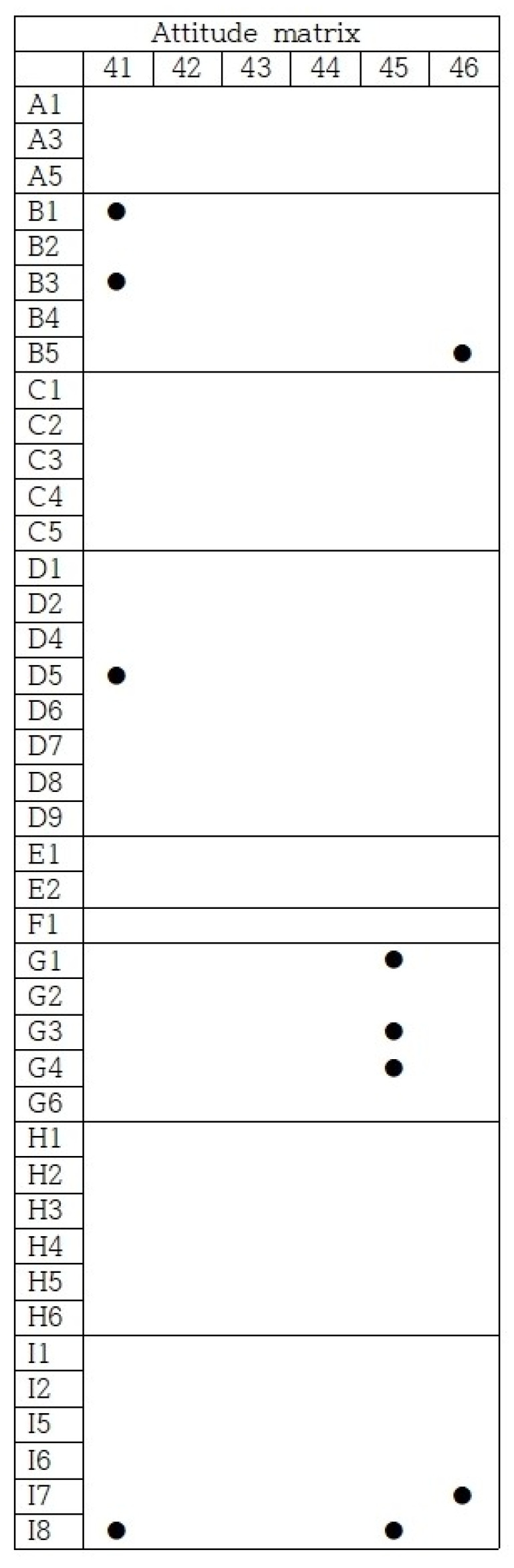
Knowledge, skills, and attitudes matrix. The black dots indicate the intersections between the KSA and DACUM charts.

The knowledge, skills, and attitudes suitable for the key tasks were converted into a matrix (Figure 1), and the subjects necessary for the core tasks were derived. The knowledge, skills, attitudes were cross-referenced against the duties and tasks identified in Table 4.

A total of 13 subjects were derived: “Understanding Counselee”, “Clinical Practice”, “Therapy-Assisting Animal Management”, “Case Conceptualization”, “Psychological Test and Evaluation”, “Program Development”, “Introduction to Animal-Assisted Interventions”, “Counseling and Understanding Psychology ”, “Evaluation Analysis and Reporting”, “Case Research and Practice”, “Case Guidance and Management”, “Training and Behavior”, and “Animal Welfare”.

### 3.5. Derivation of Curriculum Subjects

Based on the curriculum designed in Table 5, a further refinement was conducted to design the main content. Three experts from the field were involved in this task, and the results are presented in Table 5.

### 3.6. Curriculum Comparative Analysis

Using the main content outlined in Table 6 as a foundation, we conducted a thorough comparative analysis with the European Qualifications Framework (EQF) established by the International Society for Animal-Assisted Therapy (ISAAT) [27], a guideline endorsed by IAHAIO. In this analysis, we meticulously assessed the learning topics, learning outcomes, knowledge, and competencies outlined within the EQF, juxtaposing them with the core content developed through this study. This comparative approach aimed to pinpoint any divergences between the two frameworks, thereby providing insights into the strengths and potential areas for improvement in each.

## 4. Discussion

In South Korea, animal-assisted therapy was introduced in the early 2000s. However, due to a lack of data on the duties of animal-assisted therapy specialists, it has been difficult to grasp the specific responsibilities of these professionals. Therefore, in this study, the duties of animal-assisted therapy specialists were analyzed through the DACUM technique, and as a result of the job analysis, it was finally determined that animal-assisted therapy specialists have 9 duties and 53 tasks, and need 37 knowledge points (K), 32 skills (S), and 47 attitudes (A) to perform their duties.

The contents of the duties and tasks of animal-assisted therapy specialists were defined as <A. Securing a Counselor>, <B. Service User Identification>, <C. Program Design>, <D. Consultation Progress>, <E. Consultation Evaluation>, <F. Follow-Up Management>, <G. AAT Specialist Competency Development>, <H. Treatment-Assisting Animal Management>, and <I. New Program Development>. The process includes identifying the service users, conducting psychological tests, case conceptualization, collecting counseling strategies, program design, understanding the clients, selecting a program format suitable for the characteristics of the client, conducting counseling, conducting psychotherapy interventions, and performing duties covering all matters related to treatment mediators. The results are consistent with the definition [4] that therapists should focus on improving the physical, cognitive, behavioral, and social–emotional functions of a particular subject and should have knowledge of animal behavior, needs, health indicators, and stress control.

The above findings will be helpful for practitioners to accurately understand their roles and enhance their professionalism since they provide practical knowledge that can be directly applied in real-life situations by animal-assisted therapy specialists. Moreover, they will serve as guidelines for these specialists to grow into experts in the field by identifying the knowledge that is actually needed. This will play a crucial role in improving the competence of practitioners and enhancing the quality of AAT.

Using a DACUM analysis, the job analysis results for forest prenatal care instructors [23], occupations belonging to complementary and alternative medicine, were <A. Understanding Forest Prenatal Care Subjects>, <B. Cultivating Forest Prenatal Care Professional>, <C. Operation of Forest Prenatal Care Program>, <D. Self-Development> and for horticultural therapy practitioners [20], they were <A. Decide on Horticultural Treatment Implementation Institution>, <B. Diagnosis and Assessment of Horticultural Treatment Subjects>, <C. Planning Horticultural Treatment Program>, <D. Preparation for Implementing Horticultural Treatment Activities by Session>, <F. Conducting Horticultural Treatment Activities by Session>, <G. Conducting Comprehensive Evaluation of Horticultural Treatment>, and <H. Self-Development as a Horticultural Therapy Practitioner>, which can be seen as having similar structures. However, this is different from previous studies in that one of the major characteristics was the addition of management of treatment mediators, counseling evaluation for clients, follow-up management, and the development of new programs. These were derived by highlighting the importance of duties rather than belonging to tasks, and duties and tasks were subdivided according to the order and process of the job, unlike research in similar occupations. Based on the results of the job analysis, horticultural therapists were used as basic data for the standards and educational content of the qualification process, and prenatal forest healers also laid the foundation for the development of curriculum subjects through a job analysis. In the case of occupational therapists [21], the job analysis provided basic data for expanding the domestic occupational therapy area and developing and improving the curriculum. Therefore, based on this study, the current and future application plans for the job analysis results of animal-assisted therapists are as follows: to complete the responsibilities and tasks of animal-assisted therapists working in the clinical field, there is a need for continuous additional education so that current animal-assisted therapists can maintain their qualifications and improve their professional competencies considering recent trends and knowledge. Kim Soo-yeon et al. [20], after conducting a job analysis of horticultural therapists using DACUM, conducted a survey on horticultural therapists nationwide over the past decade to evaluate their current performance and future needs and analyzed their educational needs. Therefore, job performance evaluation and addressing educational needs, such as the research methodology of horticultural therapists [20], are currently considered difficult tasks because of the lack of active personnel despite numerous qualification systems (around 40) operating for animal-assisted therapy specialists. Therefore, if the number of animal-assisted therapy specialists working in the actual clinical field increases in the future, it will be necessary to revise and supplement responsibilities, tasks, knowledge, and technical attitudes through research, including surveys, to identify the actual conditions and needs of AAT specialists working in the field and reorganize them through a job analysis according to the qualification level. 

In the design of the educational curriculum, a comparative analysis was conducted with the European Qualifications Framework (EQF) of the International Society for Animal-Assisted Therapy (ISAAT). The EQF is structured in an open format to accommodate different learning needs based on the learners’ experiences and educational backgrounds. It is divided into knowledge, skills, and competencies to fulfill learning of different topics according to the learning domain. When comparing and analyzing the educational content developed in this study with the EQF, it was found that “clinical practice”, “case conceptualization”, and “psychological test and evaluation” were identified as the main additions. The fact that not only the basic theories are included, but also a certain period of clinical practice is required before engaging with clients, and that after conducting case conceptualization and psychological assessments with clients, AAT should be conducted, indicates a further advancement from traditional AAT. This seems to be a result of the evolving awareness that clients need to be analyzed more intricately before conducting AAT, and thus requiring a more specialized approach. Furthermore, the emphasis on evaluating AAI programs from the perspective of program development in the educational curriculum underscores the importance of accurately assessing whether meaningful changes occurred for clients after undergoing AAT. Another noteworthy aspect is that, in “Animal Welfare”, it is not only about overall animal welfare but students are also separately educated on the welfare of therapy-assisting animals, which serve as therapeutic aids. In AAT, therapy-assisting animals work together in a triangular relationship with AAT professionals and clients. The emergence of a separate subject dedicated to the welfare of therapy-assisting animals suggests that their welfare is deemed as important as that of humans. This underscores the significance of therapy-assisting animal welfare in AAT.

## 5. Conclusions

The results of this study may help current or prospective animal-assisted therapy specialists understand their roles and contribute to the advancement of the field. Animal-assisted therapy specialists receive training through various curricula. To enhance their competencies, improve animal welfare, and elevate the quality of therapy, tailored education for animal-assisted therapy specialists is necessary.

Through this study, as curricula are developed, there is a need to establish a framework that aligns with them. The duties, tasks, knowledge, skills, and attitudes of animal-assisted therapy specialists have been identified. Therefore, this study is significant in real-life settings by providing essential knowledge and tools for animal-assisted therapy, making it highly relevant in practical contexts. However, since it is not possible to cover all the duties of animal-assisted therapy specialists, a more detailed job analysis in the future would be beneficial.

## Figures and Tables

**Table 1:** Animal-assisted therapy specialist definition.

Animal-Assisted Therapy Specialist Definition
Expert who supports psychological and emotional recovery, social adaptation, and development of the client through psychotherapeutic intervention using human–animal interactions.

**Table 2:** Duties and tasks.

A. Securing counseling resources	A-1 Identify service user types.	A-2 Discuss the site of the consultation.	A-3 Understand the field physical Environment.	A-4 Guide service users.	A-5 Adapt therapy animals to the consultation site.
B. Check the service users	B-1 Consult with agencies and individuals.	B-2 Perform intake interview.	B-3 Prepare psychological test.	B-4 Conceptualize case.	B-5 Create counseling strategy.
C. Program design	C-1 Investigate the right program for the counselee.	C-2 Select the appropriate program format for the counselee.	C-3 Select a therapy animal and determine its duties.	C-4 Set up the physical environment in consideration of the counselee and the therapy animal.	C-5 Design the appropriate program for counselee.
D. Progress consultation	D-1 Check the consultation environment.	D-2 Check therapy animal’s condition before session.	D-3 Obtain consent after explanation to the service user.	D-4 Form relationship between the client, AAT specialist, and animal.	D-5 Perform psychotherapy intervention.
	D-6 Provide a physical environment as the situation changes.	D-7 Check for and take action on therapy animal stress signals.	D-8 Document the session process.	D-9 Consult on closure later in the session.	
E. Evaluation of counseling	E-1 Evaluate of consultation results.	E-2 Write the results report.			
F. Follow-up management	F-1 Identify the current state of affairs.	F-2 Decide whether to consult further.	F-3 Document the follow-up results.		
G. Development of AAT specialist competency	G-1 Learn up-to-date information on relevant areas.	G-2 Perform supervision.	G-3 Participate in case meetings.	G-4 Attend an academic meeting on animal-assisted interventions.	G-5 Publish the results of animal-assisted intervention cases in journals.
	G-6 Develop AAT specialist self-awareness and perform self-care	G-7 Perform self-promotion and marketing.			
H. Therapy-assisting animal care	H-1 Identify the characteristics of therapy-assisting animals.	H-2 Educate therapy-assisting animals.	H-3 Become certified as a therapy-assisting animal.	H-4 Perform veterinary management of therapy-assisting animals.	H-5 Ensure animal welfare in every process.
	H-6 Establish a management plan for the retirement of therapy-assisting animals.				
I. Development of new programs	I-1 Perform service user analysis.	I-2 Set program objectives.	I-3 Collect the preceding programs.	I-4 Analyze the preceding programs.	I-5 Develop programs that are appropriate for service user.
	I-6 Check program validity.	I-7 Execute and apply the program.	I-8 Analyze program results.	I-9 Check the significance of the program.	I-10 Modify the program.
	I-11 Review the precautions.	I-12 Document the development plan.			

**Table 3:** Knowledge, skills, and attitudes.

Knowledge
Has understanding of animals and basic knowledge of them - Knowledge of animal instincts and development - Knowledge of animal behavior - Knowledge of animal training - Knowledge of animal learning Knowledge of basic veterinary medicine - Knowledge of anatomy - Disease-related knowledge - Knowledge of animal nutrition Knowledge of pet zoology Knowledge of basic hygiene and beauty care
Knowledge of AAT animal welfare Knowledge of animal ethics and bioethics Knowledge of animal protection laws
Knowledge of educational systems and policies Knowledge of welfare policy General knowledge of animal-assisted interventions (AAIs) Knowledge of the effectiveness of AAIs Knowledge of AAI programs Knowledge of AAI objectives Knowledge of budget planning Knowledge of documentation Knowledge of anthrozoology Knowledge of recent trends in AAT Knowledge of complementary alternative medicine
Knowledge of psychological theory Knowledge of psychological practice Knowledge of counseling ethics Knowledge of case conceptualization Knowledge of protector counseling
Knowledge of abnormal psychology Knowledge of impairment characteristics Knowledge of personality psychology
Knowledge of developmental psychology
Knowledge of the client’s understanding - Knowledge of the client’s medical information - Knowledge of client’s psychological and emotional conditions - Knowledge of the client’s physical condition - Knowledge of client’s social conditions - Knowledge of AAT specialist–animal–client dynamics
Statistics-related knowledge Knowledge of evaluation methods Knowledge of how to write a thesis Knowledge of case study methods
Knowledge of psychological test Knowledge of assessment tools
Knowledge of emergency management
**Skills**
Intake interview ability Ability to analyze the client’s overall condition Ability to grasp the client’s needs Ability to gathering client information Ability to develop a treatment program Ability to construct a therapeutic environment
Counseling skills The ability to utilize animal dynamics Communication skills
Ability to understand agency operational guidelines Ability to grasp administrative procedures Ability to consult with an institution Ability to understand service regulations Ability to understand laws Ability to understand physical environment criteria
Logical reasoning ability Observational ability
Ability to search for information
Ability to work with documents Ability to use evaluation tools Ability to evaluate the effectiveness of AAT Ability to collect and analyze literature data
Ability to judge situations Discerning ability
Ability to establish an interpersonal relationship (rapport-building skill)
Animal-handling ability Ability to identify signals sent by therapy-assisting animals Ability to manage therapy-assisting animal hygiene
Self-examination and self-analysis ability Ability to manage stress and emotions
Crisis management capability
Ability to use medical accessories
**Attitudes**
Determined, cooperative
Neutral attitude, Dispassionate, Objective, Reliable
Considerate, Inclusive, Respectful, Compassionate, Patient, Has sense of humor
Careful, Detailed, Sensitive
Positive attitude, Has affinity, Receptive, Friendly, Humane, Polite
Flexible, Takes the initiative and sets an example
Creative, Curious
Enterprising attitude, Aggressive, Spirit of sacrifice, Has drive
Confident, Responsible, Sincere, Persistent, Accurate, Cautious, Prepared, Has concentration
Has sense of ethics, Has own philosophy, Professional, Clean, Has a neat appearance, Manages expression, Has academic fervor, Has a sense of purpose

**Table 4:** Subjects of core courses.

Core Course	Knowledge (K) Skill (S) Attitude (A)		Subject
A1	K	10, 11, 30	Understanding Counselee
	S	2, 3, 4, 10, 11, 12	
	A	5, 35	
A3	K	10, 11, 16, 37	Clinical Practice
	S	15	
	A	14, 35	
A5	K	1, 2, 7	Therapy-Assisting Animal Management
	S	8, 17, 27	
	A	11, 23, 37	
B1	K	10, 11, 12, 23, 25	Understanding Counselee
	S	2, 3, 4, 9, 14	
	A	2, 41	
B2	K	21, 22, 23, 25, 30	Case Conceptualization
	S	1	
	A	13, 18, 19	
B3	K	26, 30, 35, 36	Psychological Test and Evaluation
	S	2, 20	
	A	3, 35, 41	
B4	K	5, 17, 24	Case Conceptualization
	S	16, 19, 24	
	A	16, 26, 33	
B5	K	15, 22	Case Conceptualization
	S	5, 6, 7, 8	
	A	4, 32, 43	
C1	K	19, 30	Program Development
	S	2, 3, 4, 16, 18, 22	
	A	28, 33	
C2	K	14, 30	Program Development
	S	5, 6, 13	
	A	5, 14	
C3	K	2, 3, 7, 14	Therapy-Assisting Animal Management
	S	6	
	A	31, 36	
C4	K	7, 30, 37	Clinical Practice
	S	6, 8, 15, 31	
	A	1, 7	
C5	K	14, 17	Program Development
	S	16, 19	
	A	28, 35	
D1	K	12, 30, 37	Clinical Practice
	S	10, 15	
	A	14, 37	
D2	K	2, 4, 6	Therapy-Assisting Animal Management
	S	27	
	A	9, 32	
D4	K	12, 18, 22, 30	Introduction to Animal-Assisted Interventions
	S	3, 25, 27	
	A	2, 7, 23	
D5	K	12, 21, 22, 23, 25, 26, 27, 28, 29, 30	Counseling and Understanding Psychology
	S	2, 7, 8, 17, 26	
	A	38, 41	
D6	K	37	Clinical Practice
	S	23	
	A	23, 30	
D7	K	1, 2, 3, 4, 7	Therapy-Assisting Animal Management
	S	27, 31	
	A	1, 38	
D8	K	17	Program Development
	S	19, 23	
	A	5, 14, 33	
D9	K	22, 25	Counseling and Understanding Psychology
	S	2	
	A	3, 22	
E1	K	32, 35, 36	Psychological Test and Evaluation
	S	20, 21, 24	
	A	5, 32, 35	
E2	K	13, 17	Evaluation Analysis and Reporting
	S	16, 19	
	A	5, 14, 33	
F1	K	30	Counseling and Understanding Psychology
	S	2, 4	
	A	16, 26	
G1	K	18, 19, 20, 34	Case Research and Practice
	S	22	
	A	26, 34, 45	
G2	K	17, 34	Case Guidance and Management
	S	9, 29, 30	
	A	3, 18, 27	
G3	K	34	Case Research and Practice
	S	24	
	A	28, 45	
G4	K	34	Case Research and Practice
	S	22	
	A	28, 45	
G6	K	21	Case Guidance and Management
	S	29, 30	
	A	8, 9	
H1	K	1, 2, 5	Training and Behavior
	S	16, 17, 27	
	A	15, 32	
H2	K	2, 3, 5	Training and Behavior
	S	26, 27	
	A	32, 34	
H3	K	2, 3, 6, 7	Training and Behavior
	S	26, 27, 28	
	A	37, 41	
H4	K	4, 6	Therapy-Assisting Animal Management
	S	28	
	A	9, 32	
H5	K	7, 9	Animal Welfare
	S	27, 28, 29, 31	
	A	20, 39	
H6	K	7, 8, 9	Therapy-Assisting Animal Management
	S	28	
	A	9, 32, 37	
I1	K	10, 11, 30	Understanding Counselee
	S	1, 2, 3, 4, 12, 13	
	A	5, 14, 35	
I2	K	12, 13, 15, 30	Program Development
	S	24	
	A	5, 40	
I5	K	13, 14, 22, 30	Program Development
	S	5	
	A	25, 27	
I6	K	14, 32	Program Development
	S	16, 21	
	A	4, 14	
I7	K	9, 13, 22	Program Development
	S	7, 8, 23	
	A	30, 31, 46	
I8	K	31, 32	Program Development
	S	20, 21	
	A	41, 45	

**Table 5:** Curriculum and main content.

No.	Course	Main Content
1	Understanding Counselee	Abnormal Psychology
		Developmental Psychology
		Understanding Disabilities
2	Clinical Practice	Animal-Assisted Intervention Clinical Practice
3	Therapy-Assisting Animal Management	Veterinary Management of Therapy Animals
		Stress Management for Therapy-Assisting Animal
4	Case Conceptualization	Understanding Case Conceptualization
		Case Conceptualization Practice
5	Psychological Test and Evaluation	Understanding and Methods of Psychological Test
		Measurement and Evaluation of Psychological Test
6	Program Development	Animal-Assisted Intervention Program Planning
		Execution of Animal-Assisted Intervention Program
		Evaluation of Animal-Assisted Intervention Program
7	Introduction to Animal-assisted Intervention	Concept and Subcategories of Animal-Assisted Intervention
		Principles and Effectiveness of Animal-Assisted Intervention
		Understanding the Relationship between Humans and Animals
		Recent Research Trends in Animal-Assisted Interventions
		Considerations for Implementing Animal-Assisted Interventions
8	Counseling and Understanding Psychology	Group Counseling
		Personal Counseling
		Counseling Theory and Practice
9	Evaluation Analysis and Reporting	Evaluation and Analysis Methods for Animal-Assisted Interventions
		Research Methodology
10	Case Research and Practice	Analysis Techniques for Understanding Cases
11	Case Guidance and Management	Case Presentation
		Supervision
		Counselor education analysis
12	Training and Behavior	Animal Behavior
		Training and Selection of Therapy-Assisting Animals
13	Animal Welfare	Welfare of Therapy-Assisting Animals
		Animal Welfare

**Table 6:** The comparison between the developed curriculum and the EQF.

Course	Main Content	EQF	
Understanding Counselee	Abnormal Psychology	Educational, psychological, rehabilitative, and socially integrative services with animals for children, adolescents, adults, or elderly people with cognitive, social, emotional, and physical disabilities, behavioral disorders, general remedial focus and require competence enhancement.	Basic psychopathology
	Developmental Psychology		Fundamentals of gerontology and geriatrics
	Understanding Disabilities		Pedagogy specialty
Clinical Practice	Animal-Assisted Intervention Clinical Practice		
Therapy assisted animal Management	Veterinary Management of Therapy Animals	Animal health/first aid for animals	
	Stress Management for Therapy-Assisting Animals	Ethology and veterinary medicine—Relationship design	
Case Conceptualization	Understanding Case Conceptualization		
	Case Conceptualization Practice		
Psychological Test and Evaluation	Understanding and Methods of Psychological Test		
	Measurement and Evaluation of Psychological Test		
Program Development	Animal-Assisted Intervention Program Planning	Resource-oriented models	Planning and organization in the AAI environment (organizational and project management, intervention planning)
	Execution of Animal-Assisted Intervention Program		
	Evaluation of Animal-Assisted Intervention Program		
Introduction to Animal-assisted Intervention	Concept and Subcategories of Animal-Assisted Interventions	Explanatory models for AAI Definition of AAI	
	Principles and Effectiveness of Animal-Assisted Interventions	Methods for AAIs Relationships and process design in AAIs	
	Understanding the Relationship between Humans and Animals	Models of human–animal relationships	
	Recent Research Trends in Animal-Assisted Interventions		
	Considerations for Implementing Animal-Assisted Interventions	Legal requirements and aspects for AAIs Hygiene management Risk management Human first aid	
Counseling and Understanding Psychology	Group Counseling		
	Personal Counseling		
	Counseling Theory and Practice	Psychological foundations for AAIs	
Evaluation Analysis and Reporting	Evaluation and Analysis Methods for Animal-Assisted Interventions	Scientific studies on the effectiveness of AAIs	
	Research Methodology	Basics of scientific work	
Case Research and Practice	Analysis Techniques for Understanding Cases	Best practice examples of AAIs from Germany and abroad	
Case Guidance and Management	Case Presentation	Presentation and communication forms	
	Supervision		
	Counselor Education Analysis	Mental hygiene for the helping professions	
Training and Behavior	Animal Behavior	Ethological basics Animal learning behavior	
	Training and Selection of Therapy-Assisting Animals	Training of animals for use in AAIs	
Animal Welfare	Welfare of Therapy-Assisting Animals	Ethics of human–animal relationships	
	Animal Welfare		

## Data Availability

The original contributions presented in the study are included in the article, further inquiries can be directed to the corresponding authors.
